# 20. THE APPLICATION OF STEM CELL MODELS TO VALIDATE RARE AND COMMON VARIANTS CONTRIBUTING TO SCHIZOPHRENIA

**DOI:** 10.1093/schbul/sby014.079

**Published:** 2018-04-01

**Authors:** Kristen Brennand

**Affiliations:** Icahn School of Medicine at Mount Sinai

## Abstract

**Overall Abstract:** As expanding genetic studies increasingly demonstrate that both rare variants of large impact and common variants of small effect contribute to schizophrenia, it becomes increasingly critical that we unravel how these risk factors interact within and between the diverse cell types populating the brain. While mouse models are uniquely suited for demonstrating how aberrant function of single gene products contribute to aberrant neuronal function or behavior, genetic studies of penetrance and complex gene interactions are nearly impossible to address using inbred mouse lines. Similarly, the lack of human post-mortem tissue, coupled with the inability to conduct functional experiments in patient cells, has to date left us with a very limited understanding of how rare and common variants impact gene expression or cellular function. Our panelists have each developed human induced pluripotent stem cell (hiPSC)-based models for the study of predisposition to neuropsychiatric disease, establishing a new mechanism by which to systematically explore the impact of rare and common putative causal variants in human cells.

Given the heterogeneity of schizophrenia and the limited cohort sizes feasible with hiPSC-based cohorts, our panelists will share their successes and struggles in developing cohorts defined by shared clinical or genetic features. They will discuss both the molecular and phenotypic insights they have uncovered, in neurons and glia, from case/control and genetically-edited isogenic cohorts. Our discussant will focus on integrating these findings into consortia-led datasets generated from recent genomic and post-mortem studies of large schizophrenia cohorts. Our overall objective is to consider the role of hiPSC-based studies in dissecting the genetic origins of schizophrenia, validating causal variants identified through ongoing genetic analyses, and serving as a personalized medicine approach to screen for novel therapeutics with which to prevent or reverse disease course.

